# Ugandan cattle farmers’ perceived needs of disease prevention and strategies to improve biosecurity

**DOI:** 10.1186/s12917-019-1961-2

**Published:** 2019-06-21

**Authors:** Cecilia Wolff, Salvatory Abigaba, Susanna Sternberg Lewerin

**Affiliations:** 1 Department of Biomedical Sciences and Veterinary Public Health, Swedish University of Agricultural Sciences, Uppsala, Sweden; 2 Department of Production, Kabarole District Local Government, P. O. Box 38, Fort Portal, Uganda

**Keywords:** Focus group, Animal disease, Disease transmission, Livestock, Africa

## Abstract

**Background:**

Infectious diseases are an important role obstacle to high productivity in Ugandan cattle production. General disease prevention is particularly important in low-income countries, where veterinary services and adequate treatment regimens for many infectious diseases are insufficient. Improved biosecurity could lead to improved animal health and production with lower mortality and better reproductive rates. Any sustainable biosecurity interventions must be feasible for the farmers, from a practical as well as social and cultural aspect. An understanding of the farmers’ view of biosecurity and the diseases it is intended to prevent is also needed. The aim of the study was to explore the perceived needs of Ugandan cattle farmers as regards disease prevention, tools and strategies for improved biosecurity, and to assess the feasibility of basic biosecurity practices, in order to contribute to long-term strategies for improved livestock management. We conducted two rounds of focus group (FG) discussions about infectious diseases and biosecurity with cattle farmers in the districts Kabarole, Kamwenge and Kasese in western Uganda.

**Results:**

A thematic analysis revealed four organising themes: Disease prevention and biosecurity practices, Knowledge among farmers and other actors, Community and peer pressure and Services and infrastructure. From these four organising themes, a global theme of “Biosecurity is a common effort based on collective knowledge” could be derived. Diseases represent a loss of income and wealth for farmers. Lack of knowledge, training and education among farmers were seen as a challenge. While there were claims during the first round of FGs that many biosecurity measures would be impossible to practise, in all follow-up FGs at least someone had tried. Perceived barriers for implementing biosecurity were financial and cultural. Experiences that were shared were that practising biosecurity measures had made their cattle healthier, but it also incurred extra costs.

**Conclusion:**

The perceived needs of farmers that emerged include knowledge, access to veterinary services, resources and community involvement for a broader implementation of basic biosecurity. There is potential for improved cattle production by educating farmers about infectious diseases and disease prevention measures. Such training should be participatory, involve communities and encourage participants to overcome practical and cultural obstacles.

**Electronic supplementary material:**

The online version of this article (10.1186/s12917-019-1961-2) contains supplementary material, which is available to authorized users.

## Background

Uganda is a low-income country in East Africa with a great potential for developing its livestock sector [1]. As in many low-income countries, cattle production is important for agricultural economy and food security [2]. However, a high prevalence of infectious diseases is an obstacle to high productivity [3, 4]. Governmental policy as well as governmental and non-gornmental initiatives to control infectious diseases in livestock have often focused on a specific disease, usually one of importance for international trade or of immediate interest for public health, and prevention has mainly been by vaccination (if a vaccine has been available) and official movement controls [5, 6]. While such initiatives are useful, sustainable livestock production requires a broader approach to disease prevention. Given the wide range of endemic infections in cattle in Uganda [7–11], improved biosecurity in general would seem like a practical strategy for improved cattle farming.

Biosecurity, i.e. the prevention of the spread of pathogens between (and within) herds is an important factor in successful disease control in animal production [12]. It has gained increasing interest as a tool for prevention and control of infectious animal diseases at local level [13] as well as a key to reducing the use of antibiotics in livestock and hence the selective pressure for antibiotic resistance [14]. Although the main focus so far has been intensive livestock production, general disease prevention is particularly important in low-income countries, where veterinary services and adequate treatment regimens for many infectious diseases are insufficient [5, 10, 15]. Improved biosecurity could lead to improved animal health and production due to lower mortality and better reproductive rates. In addition, lower infection pressure and better within-herd biosecurity would also have a positive impact on food safety and lower the risk of direct transmission of zoonotic infections (including resistant bacteria). In a long-term perspective, better biosecurity may allow a reduction and control of diseases impacting trade [13, 16].

Low-income countries, like Uganda, have complex livestock value chains and low intensification of the livestock industry, and there is little or no stakeholder interest to invest in biosecurity. Any sustainable biosecurity interventions have to be regarded as feasible for the farmers, from a practical as well as social and cultural aspect [3, 17–20]. For subsistence farmers, even small expenditures might not be prioritised. Farmers who own cattle, at least those with a cattle herd, are rarely among the poorest but improving biosecurity still presents a challenge. It requires a change in the daily behaviour of farmers, their family and employees, and may mean that financial resources have to be reallocated. Any promotion of biosecurity is destined to fail unless there is an understanding of the farmers’ view of biosecurity and the diseases it is intended to prevent [21]. In many studies from countries with intensive production systems, veterinarians and the livestock industry are identified as players in the regulatory, social and cultural landscape farmers navigate [22–24]. In Uganda, we hypothesise that other farmers such as neighbours, family and the local community play a more important role, as animal health services and industry are less developed. Neighbours, local milk vendors or butchers buy meat, milk and eggs, but many farmers are subsistence farmers. In addition, the Ugandan population is to a large extent still rural and a third of the population is economically engaged in agriculture [25] i.e. the “landscape” is different from countries with more intensive livestock production.

With this study we wanted to learn about the farmers’ perceptions of disease, its consequences, biosecurity routines and what they perceive as possible and useful to do. Hence, the purpose was not to quantitatively estimate the knowledge, attitude and practices of farmers, but to explore their experiences, opinions and views held on cattle diseases, disease prevention and ultimately their perceived capacity to improve biosecurity.

### Aim

The aim of the study was to explore the perceived needs of Ugandan cattle farmers as regards disease prevention, tools and strategies for improved biosecurity, and to assess the feasibility of basic biosecurity practices, in order to contribute to long-term strategies for improved livestock management.

## Methods

### Study design

This study was part of a larger project on prevention of infectious disease in cattle. We used a study design where participating farmers chose the degree of intervention for their herd. Repeated focus group (FG) discussions were chosen as the data collection method because FGs are suitable for eliciting experiences and perceptions through informal discussion, and allow participants a voice in planning, implementation and evaluation of interventions [26, 27]. In this study, the topic for discussion was not sensitive and the participants were expected to have some experience with the issue. Moreover, the discussions were conducted among peers of similar socio-economic status. Focus groups made the discussions constructive and creative as opposed to what might have happened in a face-to-face interview. Another reason for choosing group discussions with farmers from the same community (area) was that this group was difficult to visit individually. In addition, when new concepts and ways of farming were introduced, ideas from one farmer might lead to others seeing it as an option as well.

### Study area

Ugandan public administration is divided into approximately 120 districts which are further divided into sub-counties, parishes, and villages. The study area was the three districts Kabarole, Kamwenge and Kasese in western Uganda. This area was chosen for the project because it is partly in the cattle corridor from the southwest to the northeast of Uganda, several different agro-ecological zones and conditions for cattle farming are present, and few studies on cattle diseases have been carried out in this area.

Each district has a district veterinary officer (DVO) who is responsible for animal health in the district. The DVOs can have several veterinary officers in their team. In addition, para-veterinarians with varying levels of training in animal health can work for the DVO office. Para-veterinarians frequently carry out treatments and give advice to farmers. Both veterinary officers and privately employed or self-employed veterinarians with a degree in veterinary medicine, as well as para-veterinarians, are by farmers commonly referred to as “veterinary doctors”.

According to the 2008 livestock census [28] there were approximately 2.8 million cattle in the study area.

### Field work

#### Recruitment of participating farmers

Participants were recruited by the local team members. They were instructed to recruit 6–8 participants, one participant per farm, per FG, and one FG per village. The sampling strategy was purposive, to gain as much information and breadth of experiences as possible from cattle farmers in the study area. The person participating in the FG should be the person responsible for cattle management. In each district the target was one FG with cattle farmers with “small farms” (up to 15 to 20 cattle), one FG with “large farms” and, if possible, one FG with only female respondents or at least 50% female participants. The villages were chosen so as to be accessible by a 4 W vehicle and known by the local vets to have farmers with an interest in improving their livestock management. In Kabarole, a fourth FG was recruited to serve as a pilot, to test and practise the methodology. The aim was to have FGs that would represent different management in terms of grazing, land-ownership, wealth of farmers, herd size and number of employees. A few of the FGs included one or two farmers who had participated in a previous study from which they knew the status of their herd regarding three endemic infectious diseases (salmonellosis, brucellosis, and bovine viral diarrhoea) and had been given basic biosecurity advice [9].

The recruitment of the focus groups/villages in Kabarole was initiated by the second author. In Kamwenge and Kasese, the first author met with the notetakers and explained the purpose of the study, how the focus group discussions and recruitment of participants would be performed, before recruitment was initiated. All local team members except the interpreter had worked with the first author earlier the same year [9, 29].

The groups were naturally occurring groups; cattle farmers with roughly the same number of cattle, as a proxy for economic status, from the same or neighbouring villages, and most of them were familiar with or knew each other. In five groups all participants were male, and there was one all-female group.

The number of FGs was decided based upon what was expected to yield saturated data without carrying out unnecessarily many FGs. In total, three groups per district were gathered. Data saturation was achieved, as noted during field work and evident during analysis.

#### The field work team

The first author, who participated during the field work, is a European veterinary epidemiologist with some limited local knowledge from carrying out field work in the study area within the same project during the year preceding the FGs. She does not speak any local language.

The second author, who was the facilitator, is the DVO in Kabarole district. The DVO is the government official responsible for e.g. animal health, animal disease surveillance, and slaughterhouse meat inspection. He has a veterinary and a master’s degree, and for farmers he is as DVO an authority in agriculture and livestock farming. He had previous training and experience in facilitating focus group discussions for participatory epidemiology.

The interpreter was a young social worker employed by the local administration in a sub-county of Kabarole. He has a Bachelor’s degree in public administration, speaks excellent English and the main local languages spoken in the study area: Lutoro and Lukiga.

In each district, the local government employed veterinarian or, in Kabarole, a privately employed para-veterinarian (bachelor in Public Health) recruited participants, and served as notetakers. They were the team members that the farmers were familiar with, and served as gatekeepers to the communities [30].

#### Set-up for focus group discussions

The FGs were carried out at a church or community hall, one was held in a participant’s home. Facilitator and participants were seated in a circle. The notetaker noted non-vocal events and the time they appeared, e.g. participants leaving or arriving, laughter, and arguing in the group. The notetaker drew a map of how participants were seated and each individual was given a reference letter (“A”, “B” and so on). The main content and context of the discussions were noted as a control of the interpreter’s translation. Referring to who said what and at what time made it possible to assess if one participant had dominated the discussion or had been very quiet. The notetaker also completed an assessment of the quality of the discussion (Additional file 1).

The interpreter made a simultaneous translation during the focus group so that the first author could follow the discussion. The first author listened, observed, and when any clarification was needed, asked questions. The translator’s voice was recorded for later transcription. The Dictaphone was not switched on until all the participants in each FG had given their consent.

The interpreter was instructed to focus on conveying what the participants meant, the “contextual meaning”, to keep language simple and if possible avoid advanced terminology (unless used by the participants).

The FGs started with the facilitator welcoming everybody and introducing the team members. All participants thereafter introduced themselves. The facilitator explained the purpose of the discussion; research about infectious diseases and biosecurity. He also explained that every member of the team, driver included, had signed a confidentiality agreement (Additional file 2) where they promised they would not discuss the FGs with anyone, not in the team and not outside the work, and that the first author would write a report from the discussions without names of villages or individual participants. It was clearly stated that anyone who felt uncomfortable or for any other reason wished to leave the FG was free to do so and that we would only voice-record the interpreter. The facilitator also explained how the notetaker would be noting things that can’t be heard on an audio recording and that we would use watches to be able to link the audio recording to notes.

The information regarding the discussion was that we were interested in the farmers’ experiences and that there were no right or wrong answers. Farmers were encouraged to ask each other questions and discuss when they didn’t agree and it was clarified that there was no need to agree within the group.

The discussion guide for part 1 (Additional file 3) had four topics. They were designed with a funnel approach, starting wide and with something expected to be familiar for all participants enabling everyone to give some input to the discussion: their experiences of (infectious) diseases in their cattle. The next topic was if and how these diseases are a problem, followed by the topic preventive measures already practised, especially biosecurity measures. Thereafter, the facilitator presented and explained graphics (figures drawn on large manila papers (Additional file 5), illustrating the transmission pathways for infectious agents between cattle. As the fourth topic the farmers’ own ideas on how to prevent disease transmission were discussed and a set of basic biosecurity measures presented (Additional file 6) and explained using illustrations. The final question for the baseline FGs was if farmers would be willing to try and practice these biosecurity measures and meet in a couple of months for a follow-up FG.

The aim of the follow-up FGs (Additional file 4) was to learn which biosecurity measures farmers had practised but, most importantly, why or why not. Farmers were asked to discuss which measures were easy, difficult and which ones they had not tried at all, if they could see benefits from practising biosecurity and, if they had not practised a measure, what would make them do this.

The main topics could be discussed in another order in case the discussion naturally took that route. Each topic had sub-issues to be covered, although not necessarily all issues in all focus groups. They were also to be used as probing or prompting questions if the discussion was slow, or to bring the discussion back to the main topic.

The participants were given a small notebook and a pen at the start of the FG. They received printed copies of the figures of disease transmission routes and the list with biosecurity measures (translated to their local language) by the end of the first FG. At some point a short break was made for refreshments. No other incentives were offered.

The baseline focus group discussions lasted from 1 to 2 h, and the follow-up FGs lasted 32 min up to 1 h and 54 min. When the discussion for data collection was finished, the facilitator opened for questions on anything veterinary-related, a way of compensating the farmers for their time and to establish trust. The session closed when the farmers had no more questions which was between 30 and 90 min in the baseline FGs but considerably shorter in the follow-up FGs.

All participants were adults. All participants who were invited to and participated in the baseline also participated in the follow-up FG, except for two. The all-female FG was the smallest with four participants at the baseline. All other groups were six to eight invited participants but a majority had attracted one or a few interested additional farmers who joined. Not all additional participants from the baseline FGs attended the follow-up FGs. In the largest FG participants were arriving and leaving, briefly or for good, so that the number of participants at one point was 17 which was too many. The facilitator struggled to keep the discussion from breaking up in smaller groups.

Before the team left the FG it was ensured that participants and the notetaker had exchanged phone numbers, for any questions the farmers might have and to be able to arrange the follow-up.

Immediately after each FG a briefing was held with facilitator, notetaker, interpreter to ensure the first author had correctly understood the main points, any disagreements and the general “tone of discussion”, and attitude from the farmers during the discussion.

The same focus group in Kabarole served as a pre-test of the discussion guide for both baseline and follow-up discussions. This also allowed for some adjustments of how facilitator and notetaker performed their roles during the FG.

### Data management and analysis

#### Transcription

The audio recordings were transcribed *ad verbatim* by two MSc students in Linguistics and English, living in Sweden but native English speakers. Because it was the interpreters’ voice that was transcribed, the length of pauses or stuttering etc. was not transcribed. The time of recording at the start of each page was noted, to be able to compare content and events to note-taker notes.

Transcripts were compared to notetaker notes to check agreement of statements and discussion.

#### Coding and reduction of data

Coding was interpretative, i.e. driven by the data, rather than pre-determined by concepts. For baseline and follow-up, respectively, four transcripts were read and a list of concepts compiled representing experiences, processes, actions, perceptions, motivations, beliefs or views, or methodological issues e.g. topics that were difficult to discuss. These concepts were organised into a coding framework of categories and subcategories (the codes). Thereafter, all 10 transcripts were read and coded using these codes.

The coded segments of text were transferred to matrices of focus groups and codes, one matrix per category, in a spreadsheet. Transcribed segments were shortened, paraphrased, and citations simplified to give a manageable volume of text while the meaning was retained.

#### Thematic analysis

The charted, condensed data were thereafter read across FGs and across codes, as well as between categories, to identify themes. The analysis followed the suggestion made by Attride-Stirling [31]. Charting of data was done in spreadsheets, then printed out to get a good overview of all data. Basic themes were identified and written on paper notes that were arranged and rearranged in the process to construct the organizing themes. In that process we discussed and repeatedly went back to the raw transcripts, and reassessed the coding framework, basic themes, and the organizing themes.

The themes identified from the condensed data are hereafter referred to as “basic themes”. From the basic themes “organizing themes” were constructed. Finally, the organizing themes were linked to a global theme.

## Results

The basic themes are presented under their respective organizing themes (the four headings below), which all include basic themes from both baseline and follow-up. The first organizing theme deals with perceptions and experiences that apply directly to cattle diseases and biosecurity practices. The following three organizing themes deal with needs and strategies in the context of how farmers keep their cattle and the landscape they have to navigate. The type and number of biosecurity measures that had been implemented, or tried, by the farmers varied between and within FGs. The text under each heading (basic theme) aims to convey what was said during the discussions.

### Disease prevention and biosecurity practices

Diseases in cattle meant a loss of income and wealth for farmers. Diseases were said to impact on their life in several ways, through the worry for finances and family, and as future prospects of a life in poverty. Losses were minimized, for example, by selling sick cattle to a butcher.

While there were concerns and claims during the first round of FGs that several of the biosecurity measures would be impossible to practise, in all follow-up FGs at least someone had tried.

The positive experiences included healthier cattle and less expenses (for treatments). Positive statements also included that it was easy to separate diseased cattle from healthy ones, easy to wash hands and asking the veterinary doctor to wash before treating cattle, and it was easy to clean borrowed spray pumps etc. It was mentioned that it is important to wash and change clothes after a visit to a market where there are sick cattle. Some of the older farmers described how they used to change clothes after visiting a cattle market, but that nowadays this is no longer practised.

In contradiction to the positive statements were those that described (sometimes the same) biosecurity measures as difficult, or impossible. The reasons that emerged were a group of themes relating to financial resources. It was said that isolating cattle was difficult, especially the “strong and walking”, due to lack of time and the extra land needed. It was mentioned how challenging it was, or would be, to wash hands and boots. Lack of resources to keep one’s own bull leads to bulls being moved between herds. Lack of time or staff to change established routines, costs for fencing material or employing (additional) herdsmen were identified as barriers. Extra costs without further specification were mentioned. Poverty was expressed as a barrier or complicating factor for improving biosecurity. Fencing off pasture was said to be “the only solution” but this requires one’s own land and financial means to buy fencing material. With no water source on one’s own land, or with limited or no own pastureland, it was perceived as difficult to keep cattle separated. One solution suggested was to reduce the herd size to be able to keep cattle only on one’s own premises. However, farmers who had their own land for pasture still found that others grazed their cattle near or even on their land.

The participants realised how the routine by all farmers and/or herdsmen of bringing their cattle to the watering point during the same short time interval each day increase the mixing of cattle from different herds. However, accessing the water point during another time of the day was perceived as something impossible. This was due to the long distance (especially during the dry season) but it was also proposed that herdsmen would refuse to change their habits.

### Knowledge among farmers and other actors

All FGs discussed the lack of knowledge among farmers and community. The lack of information, training and education (“sensitization”) for farmers was seen as a challenge.

Disease was described as something haphazard and incomprehensible, and a wish to learn more about diseases was expressed. That disease is caused by factors that are beyond the control of the individual farmer was a recurring theme. It was mentioned how animals break into the farm or are released there to graze, or how irresponsible farmers don’t look after their cattle, which leads them to stray. In addition, “others” that graze communally were said to have disease among their cattle, and people and animals that don’t belong to the community (butchers, traders, stray cattle, cattle at markets) were blamed for bringing disease.

A lack of understanding of subclinical infection and incubation periods was revealed in the perception that a vet doctor can examine an animal and see if it is not only clinically healthy but “clear of disease”. There was also the perception that purchasing cattle, or lending a bull from a friend is risk free. The logic was that when you trust the owner you would know or be able to ask about the history of the cattle, and know it has been looked after.

Poor knowledge regarding spread and transmission of infectious diseases was discussed as a cause of poor biosecurity. It was stated by the groups that “we didn’t know, but now that we have been sensitized we will practice [biosecurity]”. On the contrary, neglect to do what one knows one should do was also raised, suggesting that some farmers were not convinced they would practise biosecurity even after learning about it. It was stated that farmers who took part in the FGs had a responsibility to continue practising what they had learned about biosecurity and to share this with other farmers.

In addition to farmers’ negligence and incompetence, incompetence was also perceived as a problem for herdsmen. It was stated to be difficult to find skilled herdsmen.

A difference in skills between different types of veterinary doctors was also identified, where the professional doctors were regarded as those who wash, while non-professional doctors and those who come to treat the animals (para-veterinarians) don’t wash.

Veterinary drugs and tick-sprays are frequently used by the farmers and were perceived as something necessary, not only as treatment for disease but also as prevention, to keep cattle alive. It was stated that an injected drug or a spray clears the animal from disease momentarily, in some cases this was regarded as especially efficient if a veterinary doctor administers an injection.

Nevertheless, treatments were described as often without effect, farmers admitted that not knowing the cause of disease meant not knowing the proper treatment. Treatments were based on previous experience and advice from other farmers. The participants regarded treatments as a costly and high-risk investment, and said failures were common. Vaccines were seen only as a response to disease outbreaks.

### Community and peer pressure

Cattle farming is carried out in the context of community, culture and among peers, this was reflected in several basic themes. Farmers expressed how they learn together, help each other, and how communities are sensitized together. For example, farmers lend each other drugs when someone cannot afford to buy them or get to a drug shop. Bulls are commonly borrowed from other farmers who can afford to keep a bull. And, importantly, farmers “lend a helping hand” to each other for various matters with the cattle. Within the community, it is important to be trusted and accepted but farmers also trust their peers. This means that cattle from neighbours and friends are not isolated before mixed with your own herd.

The negative sides of community life that emerged were that jealousy within the community may rise, e.g. calling a farmer proud or as someone boasting if s(he) fences off farmland and enforces protective measures such as asking visitors to wash, or buries dead cattle.

In the FGs where farmers had their own land, it was clear that fencing off farmland creates problems. People will cut the fences to continue passing through on footpaths, especially if the footpath leads to a water source. Putting up fences was described as something that interferes with people’s lives and routines and consequences are not to be taken lightly. Further, one cannot deny other people within the community to graze their cattle on one’s land. It was mentioned how conflicts may escalate as far as to having your cattle killed.

Moreover, it was stated that one cannot ask people who come to help to wash their hands or boots/feet before reaching the cattle, as they would call you proud, call you names and, importantly, would not help you in the future. Hence, unless it is something that everybody does, this would not be feasible, one has to conform to the community. However, most preventive measures would be accepted if it was part of a cultural script. A theme was “community first” and the participants’ solution for how to bring the communities on board and include everybody in the work towards improved biosecurity was to engage the local leaders.

It was stated that, because of poverty, burying cattle carcasses is not accepted. People will dig them up and eat/sell the meat. This was repeatedly mentioned in the FGs. Examples were given of how people had been threatened for attempting to make carcasses inedible with chemicals. It was argued that selling the meat gives at least some, much needed, income even if it is smaller compared to selling the cattle to a butcher or in the market when healthy. Participants shared experiences of people having fallen ill or even died from eating dead cattle. Misconceptions within the community were mentioned, for example about how only certain organs from diseased cattle should not be consumed as food. Aborted foetuses, membranes, and dead calves were viewed differently, they can be buried. However, many farmers mentioned that dogs would find and eat carcasses and membranes before they have been buried.

### Services and infrastructure

Herdsmen, i.e. staff that look after the cattle were described as lacking in knowledge and difficult to control. If a herdsman is given an order he does not agree with, he might leave for another job. Farmers expressed how they depend on their herdsmen and struggle with herdsmen leaving for other jobs, especially if they are challenged regarding how they carry out their work.

Participants discussed how veterinary doctors are too few and too far away. It appeared that veterinarians fail to set a good example. They were said not to wash their hands or boots, not clean their equipment, and to re-use syringes. Farmers said they would not be able to ask the veterinarian to wash out of fear that he would not return.

Participants were very vocal about what they perceived as lack of support from Government, NGOs and other organisations. They talked about how farmers should be provided with affordable veterinary services with vet doctors placed locally, as well as drugs and treatment for their cattle. The vet doctor provided by the Government should not only be available but actively visit farmers to examine and monitor the cattle, sensitize the farmers and give biosecurity advice. If all farmers were continuously sensitized, they expressed how biosecurity measures they had yet not tried, or succeeded with, would be practised.

From the four organizing themes listed above, a global theme of “Biosecurity is a common effort based on collective knowledge” could be derived.

## Discussion

During the last decade, a number of studies on biosecurity behaviour, knowledge and attitudes among livestock and poultry farmers in high-income countries have been published [14, 20, 23, 24]. Studies from low-income countries or emerging economies are less frequent but are increasing [18, 32–36]. However, assessments of feasibility and implementation of suggested biosecurity practices are rare [33, 36]. Available studies from East Africa [9, 15, 37–39] suggest that biosecurity is poor. In the current study, we wanted to follow up on our previous studies on biosecurity practices and prevalence of infectious diseases in the area, to learn more about the farmers’ own perception of their needs, what they could and wanted to do as regards improved biosecurity.

It was clear that cattle diseases are perceived as a problem. The farmers’ stated needs of more basic knowledge of cattle management and disease prevention indicate a potential for improved practices via educational efforts. Farmers’ knowledge should not be underestimated [8, 37], but some knowledge gaps were clearly contributing to a risk of spreading disease, such as the lack of insight into subclinical infection. If the understanding of infectious disease is that infection is always clinically manifested, disease prevention is difficult. Hence, promotion of disease prevention would benefit from including basic facts about disease transmission dynamics.

The perception that diseases occur at random, and not linked to the farmers’ own actions or practices, is similar to what has been found in high-income countries with presumably more educated farmers [24, 40]. A certain fatalism among farmers may be created by anecdotal evidence of preventive measures that failed [40].

The wish to be educated (“sensitized”) was recurrent, but farmers in high-income countries also express a need for more knowledge [24]. The importance of local communities was clear from the discussions in the FGs. Farmers themselves talked about a community approach as a future strategy to improve biosecurity. A number of projects to improve the delivery of veterinary services have been conducted in different African countries, with varying degrees of success [41]. There are numerous challenges, but community participation appears to be crucial for sustainability [41].

Vaccination was associated with diseases that are controlled by legislation and government-funded vaccination programmes. This perception could be due to lack of available vaccines, lack of financial resources, or a lack of insight into how vaccines can be used strategically.

The results indicate that veterinary services are poorly developed, in terms of availability and affordability for farmers as well as quality of the services. The structure of the veterinary services, from local to national level, is an important aspect of animal health that is addressed by the world animal health organisation OIE [42] and requires long-term efforts [41].

In Uganda, veterinary services were previously provided free of charge by the government, and the loss of this benefit may have affected the farmers’ attitude and perception of their own responsibility. The expectations of government help are somewhat contradictory to the apparently low trust in the government’s capacity and willingness to improve farmers’ situation and give them support. It could be speculated that the Ugandan history of civil unrest may have contributed to a learned passiveness enhanced by reliance on foreign aid and development projects [43]. On the other hand, a certain passivity and expectations on government or industry to address disease prevention has been demonstrated among farmers in high-income countries, regardless of a low trust in government policy [24, 40]. This has been related to the “prevention paradox” where success is marked by a non-event and individual costs leads to population benefits that may be larger than individual benefits [17, 40].

This study confirmed that policies have to be practically feasible and accepted by farmers as relevant and culturally acceptable [3, 17–20]. Barriers to implementation of disease prevention measures were not merely financial, socio-cultural traditions prevented some farmers from e.g. asking visitors to wash their hands. Such aspects may have played an important part in the lack of implementation and/or refusal to try some suggested biosecurity measures. Cultural and practical barriers appeared to be linked, it was mentioned how you cannot keep people from crossing your land, cut fences, dig up buried animals etc. A large proportion of the human population are rural and some resources are shared. For example, reducing direct contact between different herds at watering points require acceptance and a change in behaviour by several people. Concerns about expenses, practicality and social acceptability were also somewhat similar to those voiced by some farmers in high-income countries [24].

The purposive sampling method means that the participants of this study are not representative of all farmers in the region [44]. For this explorative study we chose FG as the most relevant and practical method [26, 27, 44]. The design, with one initial FG and a follow-up, served to retain the group dynamic and maximised the amount of valid data [44] and data saturation was achieved. In addition, the intervention i.e. changing biosecurity practices, was expected to benefit from an exchange of experiences and general support from others in a similar situation [26]. With this study design and the way the discussions were conducted, the number of times a statement is mentioned does not equal importance, something mentioned once or twice can be equally important because it adds new information.

The local members of the team worked as gatekeepers to the local community of cattle farmers. Their observations as notetakers added to the interpretation of the results, and were influenced by their own beliefs and experiences [30, 45]. In addition, their roles as part of the research team may have changed their initial social position in the eyes of the participants but also meant that there was an employer-employee relationship with the project leader [30, 46].

The facilitator was crucial for the progress of the FGs and also served as a first translator, as the original discussion guide was developed in English and the facilitator moderated the discussions in local language. As a DVO, he was regarded as an important person and perhaps also as a government representative. Although the participants appeared able to discuss all aspects of the topics, we don’t know to what extent any power imbalance influenced the discussions. The presence of the European researcher leading the study may also have affected the power balance, despite the obvious need for interpretation and the observing position outside the discussion circle by the first author. A foreign, white academic is sometimes regarded as a potential financial benefactor [30]. In addition to the potential influence on what the participants chose to reveal, our own backgrounds are expected to influence our interpretation of the results [30, 44, 45]. All three authors have a keen interest in animal health, infectious disease control and biosecurity, but no expertise in sociology or human geography. We therefore focussed on the topic-related content of the discussions and may have missed some relevant anthropological aspects.

Power imbalance is a concern as regards informed consent [46] and therefore utmost care was taken to ensure that this was, as far as possible, given without any sense of pressure. In addition to the potential influence of the research team, group dynamics are expected to play a major role in the progress and outcome of the discussions. According to field notes and notetaker checklists there were one or two very dominant male participants in three of the FGs. Two of these were local leaders and others may have been reluctant to oppose them in the discussion. The facilitator was aware of the importance to try and involve all participants in the discussion, but this may not have been sufficient. The follow-up discussions took place during the political campaigns for the general elections in Uganda. In this region, the president’s governing party is strong and all local leaders who participated represented this party. It is possible that their presence made some other participants careful regarding what they expressed in terms of their own situation, but it is difficult to assess to what extent this may have affected the results. Excluding local leaders from the FGs was not possible.

There was a contradiction in several FGs between statements that “we will do” and “we can’t do” leading to doubts about what biosecurity measures that would actually be tested or implemented after the baseline discussion. There is a possibility that the farmers said what they perceived as expected of them. Another possibility is that they were just realistic about the prospects of changing management.

Limited compensation was offered to the participants. Financial compensation may create expectations and further affect the relationship between participants and researchers [46]. One farmer asked the local veterinarian for money for fuel both at the first and follow-up FG. At the follow-up meeting this resulted in a lengthy discussion as the other FG participants were clearly against being paid anything at all and claimed to appreciate being trained on how to prevent disease in their cattle. Similar appreciative views of discussions about biosecurity have been expressed by farmers in high-income countries and it is possible that this in itself has long-term positive effects [40].

There are two official languages (English and Kiswahili) in Uganda, and at least 32 distinct local languages, two of which are spoken in the study region (Lutoro and Lukiga) [30]. An obvious issue in this type of study is translation and the interpretation of what is said [26, 45]. The need for translation emphasised the language barrier, but body language and cultural aspects that may affect results and interpretation of results would still have been important even if all researchers spoke the local languages [26, 30, 45]. The meaning of vocal tones and body language vary between different spoken languages, regions and cultures. The translation focused on the context of what was said, and was hence heavily influenced by the interpreter’s own beliefs and experiences [30, 45]. The choice of project staff was limited and recruitment was mainly based on necessary skills and local knowledge. Other research from the same region has demonstrated some limited influence on the results depending on the interpreters’ own experience and background [30]. Our interpreter had no knowledge about the topic of the FGs but would be expected to have some previous conceptions about the situation of the rural population in the area. It was not possible to include backwards translation by a second interpreter. This is usually recommended [26, 30] but will still not give the “true” interpretation, as all interpretations are filtered and there may not be an equivalent meaning in both languages [30, 45]. Real-time simultaneous interpretation and an active but not leading role of the interpreter, as in our study, is what has been recommended for cross-language research [26, 30] but also means that any discussion about deeper meaning in the original language is closed at an early stage in the research [45]. The setup, with a bilingual facilitator and an audio-recorded real-time translation and refraining from using quotes or analysing exact words, aimed to moderate the cross-language challenges to validity as far as possible [26].

In summary, the results support the concept that improved biosecurity requires a complex change of several behaviours by several people. Building on existing practises, engaging entire local communities and using culturally compelling interventions are keys to success, as has been demonstrated in e.g. interventions for malaria prevention [19]. Although knowledge and awareness are important prerequisites, change is driven by perceptions of usefulness and feasibility as well as sociocultural factors such as status in the farming community [17, 20]. Group learning and participatory development of local solutions emerged as a suggestion from the FGs and would create an ownership of the issue, as has previously been identified as a way forward [20, 40]. Further to our study, in-depth research aiming to identify barriers and drivers that can be employed in the implementation of biosecurity advice [21] would be needed, as FGs are not optimal for this purpose [44].

## Conclusion

The perceived needs of farmers that emerged from this study include knowledge dissemination, access to veterinary services, resources and community involvement for a broader implementation of basic biosecurity routines. There is potential for improved cattle production in Uganda by educating farmers about infectious diseases and disease prevention measures. To have a chance of success, such training efforts should be truly participatory, involve entire communities and encourage participants to find practical ways to overcome practical and cultural obstacles.

## Additional files

Additional file 1: Checklist facilitator performance. (PDF 206 kb)

Additional file 2: Confidentiality statement research group. (PDF 220 kb)

Additional file 3: Facilitator's guide first FG. (PDF 250 kb)

Additional file 4: Facilitators guide follow-up FG. (PDF 250 kb)

Additional file 5: Figures of disease transmission routes. (PDF 167 kb)

Additional file 6: List of suggested biosecurity measures. (PDF 227 kb)

## Data Availability

Raw transcripts of FG meetings with all individual identifiers removed are available from the first author.

## Abbreviations

DVO: District veterinary officer
FG: Focus group
