# Supplementary material for: Ugandan cattle farmers’ perceived needs of disease prevention and strategies to improve biosecurity
Source: BMC Vet Res. 2019 Jun 21;15:208. doi: 10.1186/s12917-019-1961-2 (PMC6588948; doi:10.1186/s12917-019-1961-2)
Supplement: Supplementary file 1 — Checklist facilitator performance. (PDF 206 kb) [file 12917_2019_1961_MOESM1_ESM.pdf]

District ___________________ Date______________

Observation checklist

	YES	NO
Did the facilitator introduce self?		
Did the facilitator introduce the other team members?		
Were participants asked to introduce themselves?		
Was the topic introduced?		
Did the facilitator ask permission to record the discussion?		
Was the facilitator talking a lot?		
Did the facilitator encourage the participants to talk?		
Did the facilitator try to understand what the participants were saying?		
Was the facilitator judgemental about the topic?		
Did the facilitator give participant the idea that they gave the wrong or right answer?		
Were open-ended questions used?		
Were probing questions used?		
Could the facilitator deal with participants that were dominating (talking a lot)?		
Could the facilitator deal with shy participants?		

If clarification is needed, please write comment below.
