# Supplementary material for: Ugandan cattle farmers’ perceived needs of disease prevention and strategies to improve biosecurity
Source: BMC Vet Res. 2019 Jun 21;15:208. doi: 10.1186/s12917-019-1961-2 (PMC6588948; doi:10.1186/s12917-019-1961-2)
Supplement: Supplementary file 2 — Confidentiality statement research group. (PDF 220 kb) [file 12917_2019_1961_MOESM2_ESM.pdf]

2016-01-12

Confidentiality agreement for research team members

The overarching research question for this study is if it is possible to improve biosecurity in Ugandan cattle herds. This will be evaluated though focus group discussions and interviews with cattle farmers in the districts of Kabarole, Kamwenge and Kasese.

The study is led by Dr Cecilia Wolff from Swedish University of Agricultural Sciences, Uppsala, Sweden.

The information and experiences from the farmers in focus groups and interviews is confidential and must not to be shared with anyone outside of the research team. The identities of study participants and the name of villages of participants must not be shared with anyone outside of the research team.

I have read the above text and agree to confidentiality.

**Name**	**District**	**Date**	**Signature**

2016-01-12
