# Supplementary material for: Ugandan cattle farmers’ perceived needs of disease prevention and strategies to improve biosecurity
Source: BMC Vet Res. 2019 Jun 21;15:208. doi: 10.1186/s12917-019-1961-2 (PMC6588948; doi:10.1186/s12917-019-1961-2)
Supplement: Supplementary file 3 — Facilitator's guide first FG. (PDF 250 kb) [file 12917_2019_1961_MOESM3_ESM.pdf]

District ________________ Date _______

Focus group interview schedule Biosecurity in cattle herds in Uganda

**Introduction**	**Comments**
Greet everyone	Make sure everyone has got refreshments
Introduce everyone in the team	- Facilitator<br>- Dr Cecilia Wolff from Sweden and Makerere<br>- The translator for Dr Cecilia –voice recorded because we cannot write notes of everything. Only CW and Swedish colleague will listen.<br>- Note taker – will note things we can't hear on the audio recording
Let the participants introduce themselves	Does everyone know each other?<br>Are all from the same village or family?
Introduce the topic for the focus group:<br><br>The topic for this focus group is diseases in cattle and ways to improve the biosecurity on cattle herds<br><br>We are interested in Ugandan farmers' experiences.	Explain to the participants that:<br>- There is no right or wrong answer.<br>- You don't have to agree within the group.<br>- Feel free to talk to each other, exchange stories, ask questions, comment what the other participants say.
Informed consent	CW will write a report. There will be no names of people or the village name in the report.<br><br>All team members have signed confidentiality agreement and will not talk about the focus group with anyone not in the Team.<br><br>If anyone does not want to participate – free to leave the focus group at any time
**Discussion**	
**Main topic 1**	
**Can you give an example of when your cattle were diseased?**<br>- What happened, were all diseased or only a few?<br>- Were other animals in the village / neighbourhood also diseased?<br>- Had any new animals been brought to your herd or the village/ neighbourhood? Bought from somewhere else or a bull borrowed?	NOTE! For all topics try to have participants tell about their **experiences**, rather than opinions. Let participants **tell their story**, ask prompting questions only if needed.
**Main topic 2**	

District ________________ Date _______

**Are diseases in your cattle a problem for you?**<br>- Why or why not?	
**Main topic 3**	
**Can you give an example of what you do to prevent disease to spread to your cattle from other cattle?**<br>- Do you know any ways to prevent diseases to spread to your cattle from other cattle?<br><br>- What could you do to prevent diseases to spread to your cattle the ways the illustration shows?	This question is about spread of and prevention or **non-vector borne diseases** (not tick, mosquitos, flies etc.)<br><br>Show illustration of cows and infection routes.<br><br>Write down suggestions from the group on large paper.<br><br>If there are no suggestions: hand out papers with biosecurity measure check-list. Talk through each of the suggestions:<br>- Explain what is meant<br>- Could the participants try these on their own cattle herd?<br>- If not why?
**Main topic 4**	
**Can you give an example of something you would like to do to keep your cattle healthy that you don't do today?**<br><br>- Have you previously tried to do this?<br>- Do you know someone else who tried this? What happened?	Note! Non-vector borne diseases is the topic.
**Wrap-up**	
Would all participants be willing to try the biosecurity measures they suggested on their own herd for two to three months?<br>- Use the check-list<br>- For each measure note if tried this on their own farm and comments<br>-	If not done already – hand out papers with biosecurity measures check-list<br><br>Hand out pencils/pens to participants
**Thank participants**<br>- Does anyone have any questions?<br>- Repeat confidentiality from introduction, all participants OK with this?<br>- If participants agreed to try biosecurity measures agree that we contact them again in November- December to arrange a new focus group	Suggest we finish discussion<br><br>Make sure contact details to participants are saved by local team member.<br><br>Make sure participants leave feeling comfortable!
