# Supplementary material for: Ugandan cattle farmers’ perceived needs of disease prevention and strategies to improve biosecurity
Source: BMC Vet Res. 2019 Jun 21;15:208. doi: 10.1186/s12917-019-1961-2 (PMC6588948; doi:10.1186/s12917-019-1961-2)
Supplement: Supplementary file 4 — Facilitators guide follow-up FG. (PDF 250 kb) [file 12917_2019_1961_MOESM4_ESM.pdf]

# Focus group interview schedule Biosecurity in cattle herds in Uganda Part 2

| Introduction | Comments |
|---|---|
| Greet everyone | Remember to serve refreshments some time during the discussion |
| Introduce everyone in the team | - Facilitator<br>- Dr Cecilia Wolff from Sweden and Makerere<br>- The translator for Dr Cecilia –voice recorded because we cannot write notes of everything. Only CW and Swedish colleague will listen.<br>- Note taker – will note things we can't hear on the audio recording |
| Let the participants greet each other. | Were all participants here the first discussion? |
| Reminder of the topic for the focus group:<br>**The topic for this focus group is the biosecurity measures we discussed last time we met.**<br><br>We are interested in **your experience from practicing this in your own herd**. | Explain to the participants that:<br>- There is no right or wrong answer.<br>- You don't have to agree within the group.<br>- Feel free to talk to each other, exchange stories, ask questions, comment what the other participants say.<br>- |
| Informed consent | CW will write a report. There will be no names of people or the village name in the report.<br><br>All team members have signed confidentiality agreement and will not talk about the focus group with anyone not in the Team.<br><br>If anyone does not want to participate – free to leave the focus group at any time. |
| **Discussion** | |
| **1. Biosecurity measures** | |
| Use the list handed out to participants last time and go through each of the measures. | Finish this before the discussion starts.<br><br>NOTE! For all topics below try to have participants tell about **their experiences**, rather than opinions. Let participants **tell their story**, ask prompting questions only if needed. |

| | |
|---|---|
| **2. Ranking of biosecurity measures** | |
| Ranking of the biosecurity measures on the list from easiest to most challenging to practise<br>- From the farmers' experience, not their opinion.<br><br>If no one tried to practise a measure leave that one out of the ranking in a separate group. | Participatory exercise: hand out paper pieces with biosecurity measures written on them. Let the group discuss and arrange from easiest to most challenging according to their experience.<br><br>The farmers' discussion during the exercise will be very interesting! |
| **3. Discuss the ranking** | |
| **Start from the biosecurity measure ranked as easiest:**<br>- To those who practised: why did you practise this measure?<br>- Were there any benefits?<br>- What were the challenges to practise this?<br>- How did you overcome the challenges?<br><br>- To those who did not practise (if any): What would make you practise this measure?<br>- Anyone who tried to practise but gave up? Why?<br><br>Repeat these discussion questions for each biosecurity measure. | The easiest measures will be practised by several (hopefully)<br><br>The more difficult by only a few farmers.<br><br>Make sure that there is input from both those who practised and those who didn't for each measure.<br><br>Keep discussion focussed around these measures.<br><br>If the discussion take long time –move on to the last. |
| **4. Biosecurity measures not practised by anyone** | |
| **Discuss why not practised**<br>- What happened?<br>- What were the challenges?<br>- Can you see benefits?<br>- What would make you try this measure in your herd? | - No judging of farmers who did not practise! |
| | |
| **Wrap-up** | |
| **Thank participants**<br>- Does anyone have any questions?<br>- Repeat confidentiality from introduction, all participants OK with this?<br>- Has everyone got contact details to the local veterinarian if there are questions later? | Suggest we finish discussion.<br><br>Make sure contact details to participants are saved by local team member.<br><br>Make sure participants leave feeling comfortable! |
