# Supplementary material for: Ugandan cattle farmers’ perceived needs of disease prevention and strategies to improve biosecurity
Source: BMC Vet Res. 2019 Jun 21;15:208. doi: 10.1186/s12917-019-1961-2 (PMC6588948; doi:10.1186/s12917-019-1961-2)

Routes for spread of infectious diseases between cattle

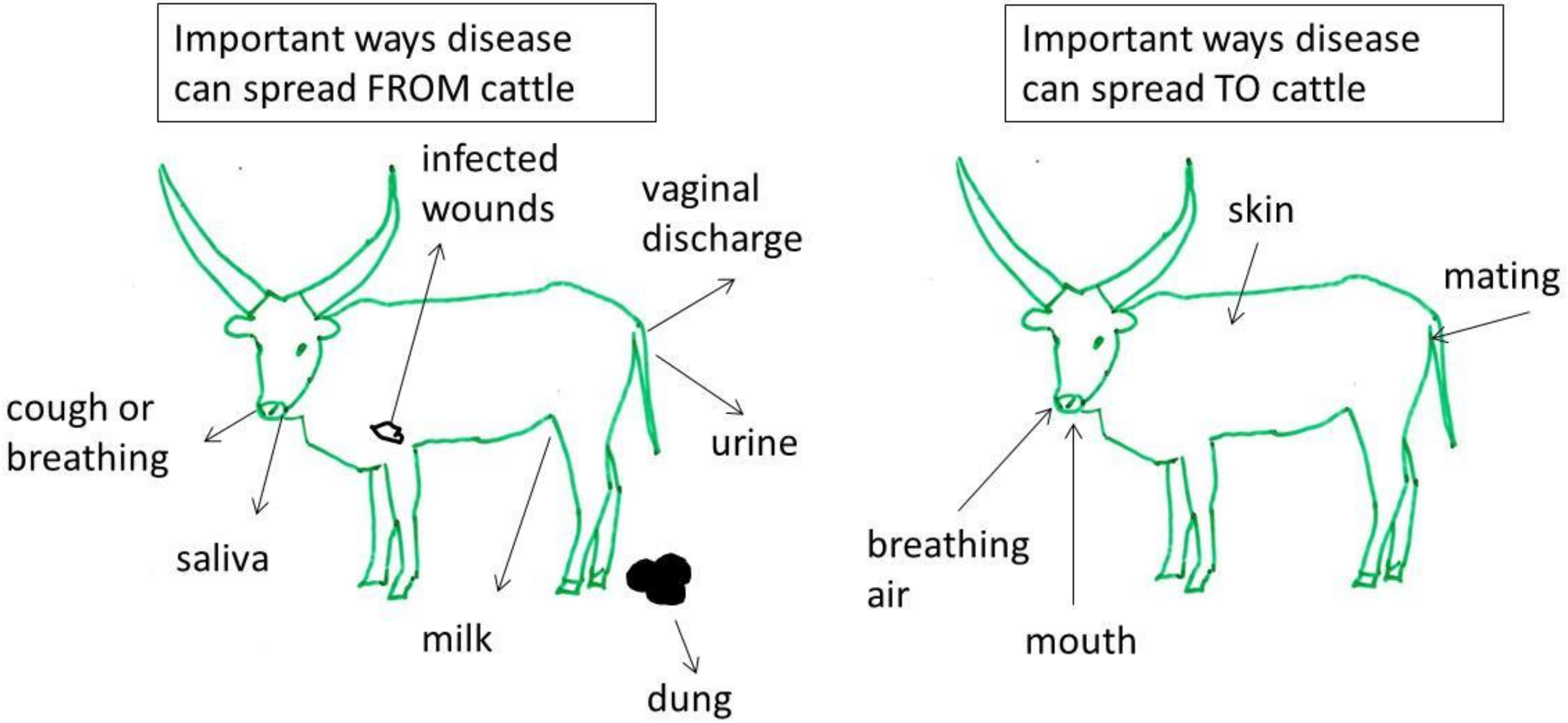

Supplement: Supplementary file 5 — Figures of disease transmission routes. (PDF 167 kb) [file 12917_2019_1961_MOESM5_ESM.pdf]
