# Supplementary material for: Ugandan cattle farmers’ perceived needs of disease prevention and strategies to improve biosecurity
Source: BMC Vet Res. 2019 Jun 21;15:208. doi: 10.1186/s12917-019-1961-2 (PMC6588948; doi:10.1186/s12917-019-1961-2)
Supplement: Supplementary file 6 — List of suggested biosecurity measures. (PDF 227 kb) [file 12917_2019_1961_MOESM6_ESM.pdf]

There are several measures you can take to reduce the risk of infectious disease to spread to your cattle, these are some examples that you may want to try on your farm

Measure to reduce the risk of disease in your cattle	Did you try this on your cattle farm?<br>If you tried this measure - was it difficult or easy?<br>If you did not try this measure please explain the reason why.
Disease can spread if your cattle meet or mix with cattle from other herds on pasture or in the village. Keeping your cattle separate from other herds reduces the risk for disease.	
Disease can spread if your cattle meet or mix with cattle from other herds on pasture or in the village. Keeping your cattle separate from other herds reduces the risk for disease.	
If you share pasture with other farmers or use communal grazing, keeping this bigger group of cattle is separate from other cattle reduces the risk for disease spread.	
At the cattle market a lot of cattle from many destinations mix and diseases are easily spread. To bring cattle to the market and return them to your herd again or to buy cattle from the market can bring disease to your cattle.	
If you buy cattle or borrow a bull you can reduce the risk for diseases to spread if you only bring healthy-looking cattle to your farm. If they are kept separately for at least a couple of days, but 3-4 weeks is better, before you put them together with your own cattle the disease risk is reduced.	
If you have handled animals that are not yours, washing your hands and cleaning boots or feet before handling your own cattle, e.g. milking, spraying, feeding, reduces the risk for disease spread.	
People that handle your cattle can spread diseases from other cattle they have met. It is good if they also wash hands and clean boots or feet before handling your cattle	

**Measure to reduce the risk of disease in your cattle**	**Did you try this on your cattle farm?**<br>**If you tried this measure - was it difficult or easy?**<br>**If you did not try this measure please explain the reason why.**
Equipment that is shared or borrowed from other farmers, like spray pumps or ropes, can spread disease. If they are cleaned before you use them on your own cattle disease risk is reduced.	
If an animal is not healthy removing it from the herd and keeping it separated until healthy again reduces the risk for disease to spread. Also washing your hands and cleaning boots, feet and equipment after handling this animal reduces risk for disease to spread.	
Dead cattle and aborted calves (and their membranes) can also spread diseases so removing this from your herd to burn or bury in the ground reduces disease risk.	
Your own suggestion:	
Your own suggestion:	
Your own suggestion:	
Your own suggestion:	
Your own suggestion:	
Your own suggestion:	
